# Picoliter droplet array based on bioinspired microholes for in situ single-cell analysis

**DOI:** 10.1038/s41378-020-0138-2

**Published:** 2020-05-18

**Authors:** Lin Du, Huan Liu, Jia Zhou

**Affiliations:** 1 State Key Laboratory of ASIC and System, School of Microelectronics, Academy for Engineering & Technology, Fudan University, Shanghai, 200433 China; 2 Tian Jin Tuo Rui Med Technology Co., Ltd, Tianjin, 200438 China

**Keywords:** Engineering, Physics

## Abstract

The division of aqueous samples into microdroplet arrays has many applications in biochemical and medical analysis. Inspired by biological features, we propose a method to produce picoliter droplet arrays for single-cell analysis based on physical structure and interface. A 0.9 pL droplet array with an RSD (relative standard deviation) less than 6.3% and a density of 49,000 droplets/cm^2^ was successfully generated on a PDMS chip (polydimethylsiloxane) from a micromachined glass mold. The droplet generation principle of the wetting behavior in the microholes with splayed sidewalls on the PDMS chip by liquid smearing was exploited. The feasibility of the picoliter droplets for bacterial single-cell analysis was verified by the separation of mixed bacteria into single droplets and isolated in situ bacteria propagation.

## Nanotechnology: Bio-inspired approach generates picoliter droplets for single-cell analysis

Researchers in China have developed a method to rapidly and reliably separate a solution into an array of picoliter-scale droplets. A team led by Jia Zhou of Fudan University first made a negative image of the surface of a lotus leaf. This served as the basis for a micromachined glass mold which was then used to manufacture PDMS chips. The structure of these chips—a series of microholes with angled walls—causes liquid smeared across the surface to form droplets within the holes. The droplets have an average volume of 0.9 pL. The team tested their approach by using it to isolate single-cell droplets of bacteria from a mixed solution. The ability to rapidly, reliably, and affordably generate picoliter microdroplets will prove valuable for studies in single-cell genomics and proteomics, as well as other fields.

## Introduction

The compartmentalization of aqueous samples into millions of independent microdroplet reactors has revolutionized chemistry and biology by enabling single-cell analysis^1,2^, high throughput screening^3^, and molecular diagnostics^4–6^. Shrinking the droplet size minimizes the consumption of precious samples (such as rare mutant alleles and noncultivable bacteria)^7,8^. For a given volume of sample with a smaller droplet size, the much greater number of droplets improves the statistical significance of the experimental results. With a few exceptions^9–13^, most microdroplet generators are continuous microchannels^14–17^, sealed chamber chips^18^, and chemically prepatterned surface chips^19,20^. Continuous microchannel and sealed chamber chips produce highly monodispersed droplet streams but require sophisticated precision pumps and surfactants to prevent coalescence^21,22^. The performance of droplet production may vary with the precise conditions of the equipment and the parts included in the equipment, such as sealed chips with necessary extra bonding or sealing. Compared with the above methods, the formation of droplet arrays on chemically prepatterned surfaces provides a high density and open structure with great potential for high-throughput applications, relying on a surface treatment to alter contact angles^23^. However, such treatment complicates the manufacturing process and increases the chemical complexity of the chip. As the droplet size decreases, the surface energy becomes more substantial, so reinforced treatments are required to form droplets below the picoliter range^24^.

Consequently, Jackman^25^ and Thalladi^26^ fabricated large arrays of microwells with a simple physical structure (without chemical surface treatment)^27–29^ on polydimethylsiloxane (PDMS) and formed 2D arrays of microdroplets for genome sequencing. Embedding the liquid samples into these microwell arrays, however, adds further complications to experimental manipulations, such as requiring a bulk solution. From the angle of minimizing sample volume, such a loading method in the single-side-opened cylindrical microwells is not well matched to the actual biological demands. The issue hinders the potential for future applications requiring a minimum sample for rapid distribution of small, uniform volumes of solutions or suspensions into spatially well-defined microreactors. Herein, we report a rapid generation method of picoliter droplet arrays based on physically structured microholes, inspired by a reversed mold from a lotus leaf. The picoliter droplet array was applied for in situ bacterial propagation for single-cell analysis.

## Results and discussion

**Fig. 1:**
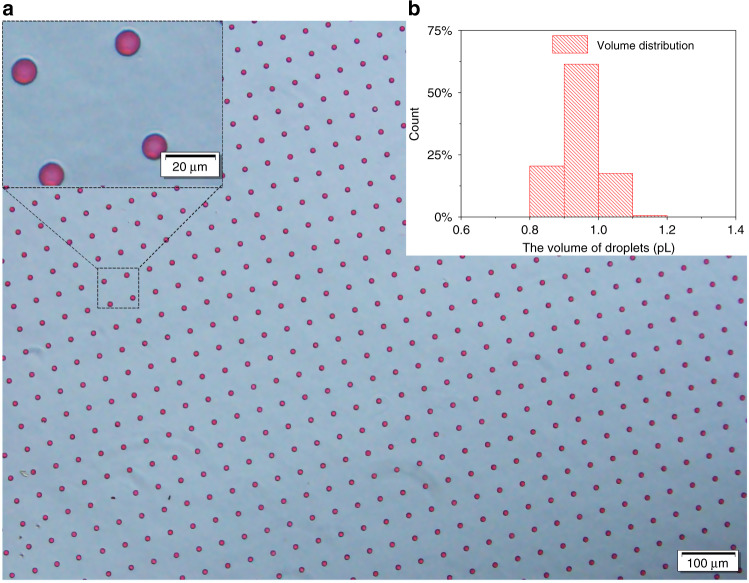
Production of picoliter droplets using a glass chip. **a** The full view under optical microscopy and partial enlargement observation of the droplet array. **b** Droplet size distribution estimated from the image

Inspired by the lotus leaf mold, we successfully developed a microdroplet array using a glass mold. The droplet array is shown in Fig. 1.

The distribution of the microdroplet array formed from the glass chip is shown in Fig. 1a. Compared with the distribution of droplet volume from the lotus chip, it is much narrower and indicates that approximately 60% of the droplet volume ranges from 0.9 to 1 pL, as shown in Fig. 1b. Unlike using cylindrical microwells, we observed the reliable formation of the microdroplet array (see Supporting Information, video) followed by the smearing process. The size of such a microdroplet array, with the relative standard deviation (RSD) of the volume smaller than 6.3%, demonstrates that the volume of microdroplets smearing from the glass chip approaches homogeneity.

**Fig. 2:**
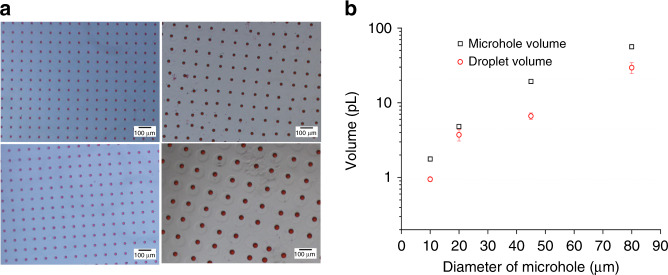
Influence of the size of the microhole on the volume of the microdroplets. **a** Detailed observation of the microdroplets in the microholes (the bottom diameters of the microholes in the glass chip were 10, 20, 45, and 80 μm) after adding mineral oil. **b** The repeatability of the microdroplets in the different sizes of microholes estimated from the image analysis

Glass molds with different sizes of microholes (10, 20, 45, and 80 μm in bottom diameter) with similarly splayed sidewalls were studied to improve the controllability of the microdroplet array. The gaps between the microholes of the glass chip in the top edge were all 35 μm, and the depth of etching was approximately 10 μm. Figure 2a shows pictures of the droplet array from each glass chip. The corresponding relationship between the different diameters of the microholes on the glass chip and the volume of the smearing droplet is shown in Fig. 2b. The volumes of droplets produced in the different diameters of microholes on the glass chip are 0.9, 3.7, 6.7, 29.5 pL, which increases with the increasing diameter nonlinearly within this range.

The mechanics of droplet formation in bionic microholes with splayed sidewalls can be discussed as follows.

**Fig. 3:**
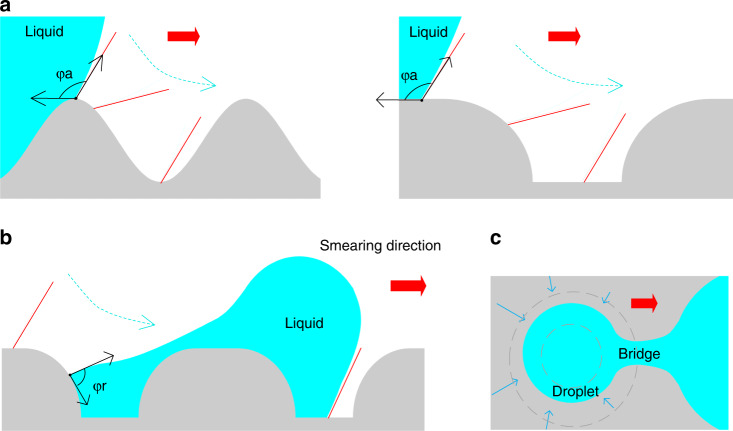
Schematic illustration of the droplet forming mechanism. **a** The wetting behavior on the corrugated microholes with the splayed sidewall. **b** Schematic cross-section of the droplet formation process on the glass chip. **c** Top view of the droplet formation process

As shown in Fig. 3, φ_a_ and φ_r_ represent the advancing angle and the receding angle on the surface of the PDMS, respectively. The previous wettability characterization shows that the angle φ_a_ is much larger than the angle φ_r_^22^. The direction of the red wide arrow in the diagram is the smearing direction. The red line indicates the contact line of the liquid at different times during smearing. The dotted arrow indicates the movement tendency of the liquid. The direction of the solid arrow indicates the direction in which the droplet shrinks.

Different from those microholes with cylindrical walls, the glass chip featured splayed microholes. On the one hand, when smearing through the splayed holes (Fig. 3a), the air effectively escapes from the bottom of them, significantly improving the filling efficiency of the holes. On the other hand, when liquid recedes from the splayed sidewall, the corrugated surface results in an altered contact line of the droplet. The contact line continues to move, and it forms a narrow bridge between the microhole and the bulk aqueous solution, as shown in Fig. 3b. Finally, the narrow bridge is ruptured to form a separated microdroplet (see Fig. 3c). The sidewall of the bioinspired microhole has the same effect as the chemically prepatterned surfaces to pin the droplets to the three-phase contact line^30,31^, as seen in Supporting Information, Fig. S2.

After comparing the maximum radius of the droplets from the top view of the glass chip, we also found that a larger radius (*R*) of the microhole is associated with a larger droplet volume. According to the geometric analysis (Supporting Information, Fig. S3), we estimated the volume of the droplet stayed in different diameters of microholes with and without considering the effect of the receding angle on the splayed sidewall, as shown in Table 1.

**Table 1:** The results of the experimental and estimated droplet volumes

Chip	1	2	3	4
The diameter of the microholes	10 μm	20 μm	45 μm	80 μm
Experimental volume (pL)	0.9	3.7	6.7	29.5
Without considering the receding angle (pL)	0.6	1.9	**8.7**	**26.6**
Considering the receding angle (pL)	**1.0**	**3.2**	14.1	42.3

The black font datum is the most accurate estimation of the volume with the multiple methods

It is clear that the receding angle is of great significance in defining the volumes of the droplet from the glass chips featuring diameters and heights close to the lotus chip (chips 1 & 2), while it has little effect on those from glass chips with larger aspect ratios of the microholes (chips 3 & 4). This can be attributed to the large diameter, which means a large distance between the two splayed sidewalls of the microholes so that the influence of the receding angle on the splayed sidewall becomes small. Regardless, the volume of the microdroplets increases with increasing radius of the microholes. However, we also observed in our experiments that the microdroplet array generated in the glass chip with a diameter of 80 μm was occasionally unstable, i.e., exhibiting small satellite droplets (Supporting Information, Fig. S4). Such results show that further study of the aspect ratio of the splayed microholes is necessary to obtain stable and reliable larger droplets volumes (~>30 pL).

Considering the differences of the microholes with splayed walls and straight walls, i.e., cylindrical wells, droplet formation using glass chips with a bottom diameter and depth of 10 μm for both was compared. The results demonstrated that the droplets could be stably generated in 100% of the 736 splayed microholes using chip 1, as shown in Fig. 1a. However, only liquid films were formed on the surface of cylindrical holes, and more than 40% of them broke within 8 s, which means that at least 40% of such cylindrical holes could not be filled with liquid by smearing. The liquid “film” actually cracked above the cylindrical microwells (similar behavior was also observed by other studies^32^). Please refer to Fig. S5 (Supporting Information). The main difference between cylindrical and splayed holes is that in cylindrical holes, the liquid forms an unstable film that quickly ruptures, whereas the splayed holes yield stable droplets. Such results demonstrate that droplet formation on cylindrical wells is not stable by smearing. Similar results were also reported by ref. ^25^.

A preliminary study of the repeatability of droplet forming using glass chips was also carried out. In our previous research, we found that chip 1 (with 10 μm as the bottom diameter of the microholes, similar to the microstructure of the bulges of the lotus leaf) showed optimum behavior to form a droplet array. Therefore, we used 10 chip 1 to show the level of repeatability in the drop size of the approach. All ten chips could produce droplet arrays well. When the velocity of smearing ranged from 0.97 to 1.39 mm/s, the average droplet volume was 1.15 pL using three chips, with an RSD of approximately 7.45%. The droplet sizes ranged from 0.9 to 6.1 pL at speeds from 0.08 to 2.15 mm/s. Such results indicate that further study of the effect of the velocity of smearing on the coefficient of variation of droplet volume is necessary.

The controllable and rapid formation of a microdroplet array is significant for quantitative particle separation^33,34^. We focused on the application in single-cell analysis, which has enormous potential in various fields, such as single-cell sequencing, cell-based detection assays, and protein expression, as well as microsensors^35^. However, facile separation of the single-cell level array from the cell culture medium is still a challenge at present^36^. In this paper, single-cell separation experiments were performed on *S. aureus*. Moreover, *E. coli* was added to the test sample to demonstrate the single-cell separation and in situ propagation in the case of mixed strains.

**Fig. 4:**
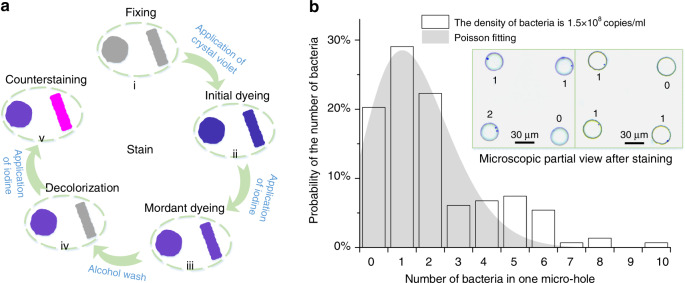
The process of staining and the statistical results of single bacterial separation. **a** Stain for *S. aureus* and *E. coli*: (i) Fixing the bacteria on the surface of the microhole in the glass chip. (ii) Initial staining for both bacteria. (iii) A mordant in the staining of *S. aureus*. (v) Decolorization of *E. coli*. (v) Counterstaining for *E. coli*. **b** The statistical results of the distribution of bacteria in one microhole; the droplets are prepared by a glass chip with a microhole diameter of 10 μm. Red bars represent experimental results; gray bars represent the fit to the Poisson distribution (*R*^2^ = 0.863)

Through the staining process (Fig. 4a), we obtained the single-cell array on the glass chip (Fig. 4b). The number distribution of bacteria suggests that approximately 29% of the microholes contain only one bacterium (see Supporting Information, Fig. S6). The isolated bacteria in the droplet array were cultured in situ without mutual interference from the surroundings for further individual research^36,37^.

**Fig. 5:**
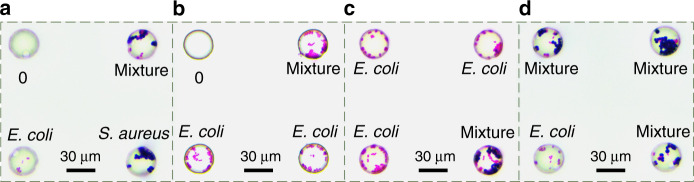
Number of *S. aureus* and *E. coli* in the microholes after propagation in situ for 20 h. **a**–**d** Morphology and quantity of two bacteria in different microholes. The purple ball is *S. aureus*, and the pink rod is *E. coli*

Figure 5 demonstrates typical examples of single-cell proliferation utilizing the smearing of cell culture medium with mixed strains. It can be seen that there are mixed bacteria (those labeled with “Mixture”), nothing (those labeled with “0”), and E. coli and S. aureus in the microholes. Such results indicate that these isolated bacteria have been successfully proliferated for several generations, increasing the number of the same species for more biological applications. For example, the individual differences in bacteria may cause disequilibrium in the microholes, which is also one of the important concerns of biologists for exploring the differences between cells. Due to the Poisson distribution, we obtained not only isolated single bacteria but also a combination of multiple bacteria. Moreover, the randomly distributed combination of bacteria can bring greater convenience to the interaction relationship between high-throughput bacteria of various groups, which is one of the most attractive topics in biology^38^. Therefore, the rapid generation method of picoliter microdroplets based on the simple physical interface is facile, sample-effective, and low-cost, which will be of great potential for the development of microdroplet arrays for biological analysis, such as single-cell genomics studies, aptamer selection, and nucleic acid analysis. The droplets produced by our alternative, simple methods are unconventional and can be implemented without using external, bulky laboratory devices such as pumps^39^. This method provides a new technical means for chemists, biologists, medical scientists, etc. to work in a wider range of applications.

## Discussion

Inspired by a reversed mold from a lotus leaf, we proposed a method based on the physical structure for picoliter microdroplet generation and application in single-cell analysis, which avoids the complicated chemical treatment of the chip surface and complex operating equipment. In the experiment, by adjusting the size of the microholes with splayed walls, we successfully obtained a microdroplet array with volumes from 0.9 to 29.5 pL. The micromachined layout ensures an accurate droplet position and arrangement. We preliminarily generated a single bacterium array from a bulk aqueous solution containing multiple bacteria and propagated the bacterium in situ. Staining of the isolated bacteria showed that they had been successfully proliferated for several generations, indicating that the method has excellent biocompatibility for biological analysis, combinatorial chemistry, and related research. The presented strategy based on the rapid molding of the simple physical structure is simple, feasible, and sample-effective for tiny microdroplet generation.

## Materials and methods

### Materials and equipment

Fresh lotus leaves were picked in the pond during midsummer (Shanghai, China). Sylgard prepolymer and curing agent (Sigma-Aldrich) were used to fabricate the PDMS molding chip. The UV-curable adhesive NOA 81 (Norland, NJ) was utilized for mold reproduction from lotus leaves under UV light (excitation maxima of 493 nm). Aqueous red ink (Ruwen, Shanghai, China) was used as a divided liquid to intuitively exhibit the results of droplet formation. Drops of mineral oil (M8410, Sigma-Aldrich, MO) were smeared by a coverslip (10212424 C, CITOGLAS, China) to prevent droplet evaporation. *Staphylococcus aureus* (29213, ATCC, VA) and *Escherichia coli* (25922, ATCC, VA) were chosen for verification of single-cell isolation and in situ propagation. Nutrient broth medium (CM106-01, Beijing Land Bridge Technology Co., Ltd, China) was provided for the rapid growth of the bacterial cells. The staining kit (BA4012, Baso Diagnostics Inc., China) was applied for intuitive observation of bacterial propagation. The glass mold with controlled-size micropillars manufactured by DongCheng Microfluidics Co., Ltd. (Zhenjiang, China) was used as the mold to reproduce the PDMS chip instead of the NOA 81 mold in most experiments in this study. The structure of the PDMS chip was investigated under SEM (scanning electron microscopy, G2 Pro X, Phenom-World, Netherlands) and 3D microscopy (VHX-600E, KEYENCE, Japan). A charge-coupled device (CCD) camera (DP73, Olympus, Japan) was used to monitor droplet formation and bacterial propagation. The image was processed with ImageJ (https://imagej.nih.gov/ij).

### Generation of picoliter droplet array

After analysis of the picoliter droplet array generated from a piece of lotus leaf, a PDMS chip reversed from a micromachined glass mold with a structure mimicking that of a lotus leaf was proposed.

#### Picoliter droplet array from lotus leaf

**Fig. 6:**
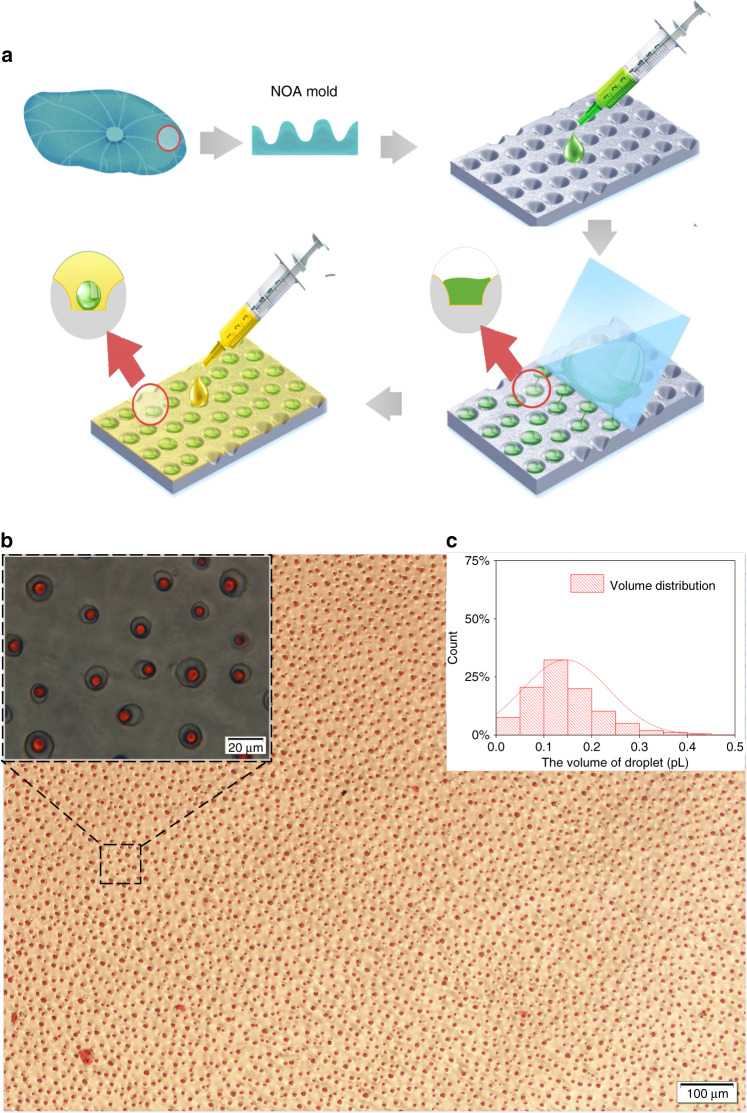
Schematic illustration of droplet forming and characterization of the picoliter droplet array. Gray, yellow, and green represent PDMS, oil, and liquid, respectively. **a** The process of droplet formation from the lotus chip: NOA molding, smearing aqueous solution and adding mineral oil. **b** The full view under optical microscopy and partial enlargement observation of the droplet array. **c** Droplet size distribution estimated from the image

A typical replication process^40^ was applied to obtain a reproducible PDMS molding chip from a piece of lotus leaf, as shown in Fig. 6a. First, a 20:1 mixture of Sylgard prepolymer and curing agent was poured on a fresh lotus leaf to make a first negative replica. Then, we obtained a positive hardened replica by curing NOA 81 from the first negative replica. Ultimately, this NOA mold was used to manufacture many PDMS chips, namely, lotus chips, for the following experiments.

Smearing is a simple and fast way to handle droplets^41,42^, as shown in Fig. 6b. The lotus chip was first placed on a clean coverslip and showed exceptional adhesion even without glue. After adding the aqueous solution on the surface of the lotus chip, droplets were formed by prompt smearing on its surface using another coverslip. Then, the newly compartmentalized microdroplets were shrunk by dripping several drops of mineral oil on the preparation, which prevented the evaporation of the droplets. The oil also washed away the excess solution remaining on the surface of the lotus chip and reduced the specific surface area of the aqueous samples. Therefore, we idealize the droplets as spheres to approximate the volume^43^. The radius (*r*) of the droplet profile was measured under a microscope. The volume (*V*) of the droplets was calculated by the formula of a sphere, i.e., *V* = (4*r*^3^)/3. Finally, a third coverslip was placed over the preparation to provide additional protection against evaporation and airborne contamination.

Figure 6c shows that the microdroplets are scattered randomly across the lotus chip. The distribution of droplet volume is wide and indicates that approximately 30% of the volume of the droplets ranges from 0.1 to 0.15 pL (Fig. 6d).

#### Picoliter droplet array from a micromachined glass mold

**Fig. 7:**
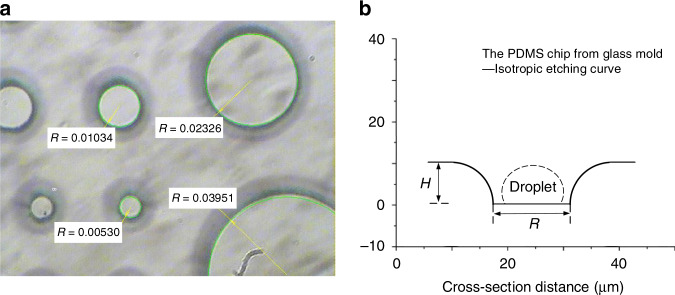
Schematics of the biomimetic glass mold and chip. **a** Picture of the glass mold with micropillars. **b** The cross-section profile of the microhole in the glass chip

To further increase the controllability of the microdroplets and significantly promote promising applications, micromachining would be one of the feasible approaches. Through characterization by SEM and 3D microscopy, we obtained the surface morphology and cross-section of the lotus chip (see Supporting Information, Fig. S1). Unlike the cylindrical microwells proposed by Jackman et al.^25^, the lotus chip exhibits a corrugated array of smooth microholes. After fitting the cross-sectional profiles, the concrete microhole has a diameter of approximately 20 μm and a depth of approximately 10 μm. Therefore, we replaced the NOA mold with a micromachined one, i.e., a piece of glass with etched micropillars, which imitated the lotus leaf with the specific feature of a gradually larger diameter from bottom to top of the microbulges, i.e., splayed walls. The fabrication process of the PDMS chip from the glass mold, namely, glass chip, was the same as for the NOA mold. The schematic of the micromachined glass mold and the glass chip is illustrated in Fig. 7. Glass chips were applied to produce droplet arrays in later experiments, if not specially defined. *H* and *R* represent the depth of etching and the bottom radius of the microhole, respectively. The process of forming microdroplet arrays using the glass chip is similar to the lotus chip, as shown in Fig. 6a.

### Separation and propagation of a single bacterium

We performed a single-cell separation experiment using *S. aureus* at a suitable concentration of approximately 1.5 × 10^8^ copies/ml according to the McFarland Equivalence Turbidity Standard. We used the nutrient broth medium for propagating the two bacteria strains in a 37 °C incubator for 20 h. To exhibit the results of single-cell analysis intuitively, we stained the bacteria without adding oil. The most significant difference from the normal staining process was that before each step of the staining, the glass chip needed to be baked to accelerate the evaporation of deionized water and facilitate the reentry of the stain into the microholes more effectively. We applied the glass chip in single-cell analysis, where the bacteria were isolated and trapped in the microholes. Additionally, we used *S. aureus* and *E. coli* to verify the picoliter droplets for single-cell analysis under non-single-species conditions.

## Supplementary information

Videos about the process of microdroplet array formation

Supporting Information

## Data Availability

The data that support the finding of this study are available from the corresponding authors on reasonable request.
