# Supplementary material for: Picoliter droplet array based on bioinspired microholes for in situ single-cell analysis
Source: Microsyst Nanoeng. 2020 May 18;6:33. doi: 10.1038/s41378-020-0138-2 (PMC8433318; doi:10.1038/s41378-020-0138-2)
Supplement: Supplementary file 2 — Supporting Information [file 41378_2020_138_MOESM2_ESM.pdf]

Supporting Information

Picoliter droplet array based on bio-inspired micro-holes for in-situ single-cell analysis

Lin Du,† Huan Liu,‡ and Jia Zhou,†*

† ASIC and System State Key Lab, School of Microelectronics, Academy for Engineering & Technology, Fudan University, Shanghai 200433, China

‡ Tian Jin Tuo Rui Med Technology Co., Ltd, Tianjin 200438, China.

*E-mail: jia.zhou@fudan.edu.cn

1. Characterization of the lotus chip

The surface morphology and cross-sectional measurement of the lotus chip were characterized by SEM (Figure S1a) and 3D microscope (Figure S1b). Moreover, the lotus chip has a corrugated array of smooth micro-pores (Figure S1c).

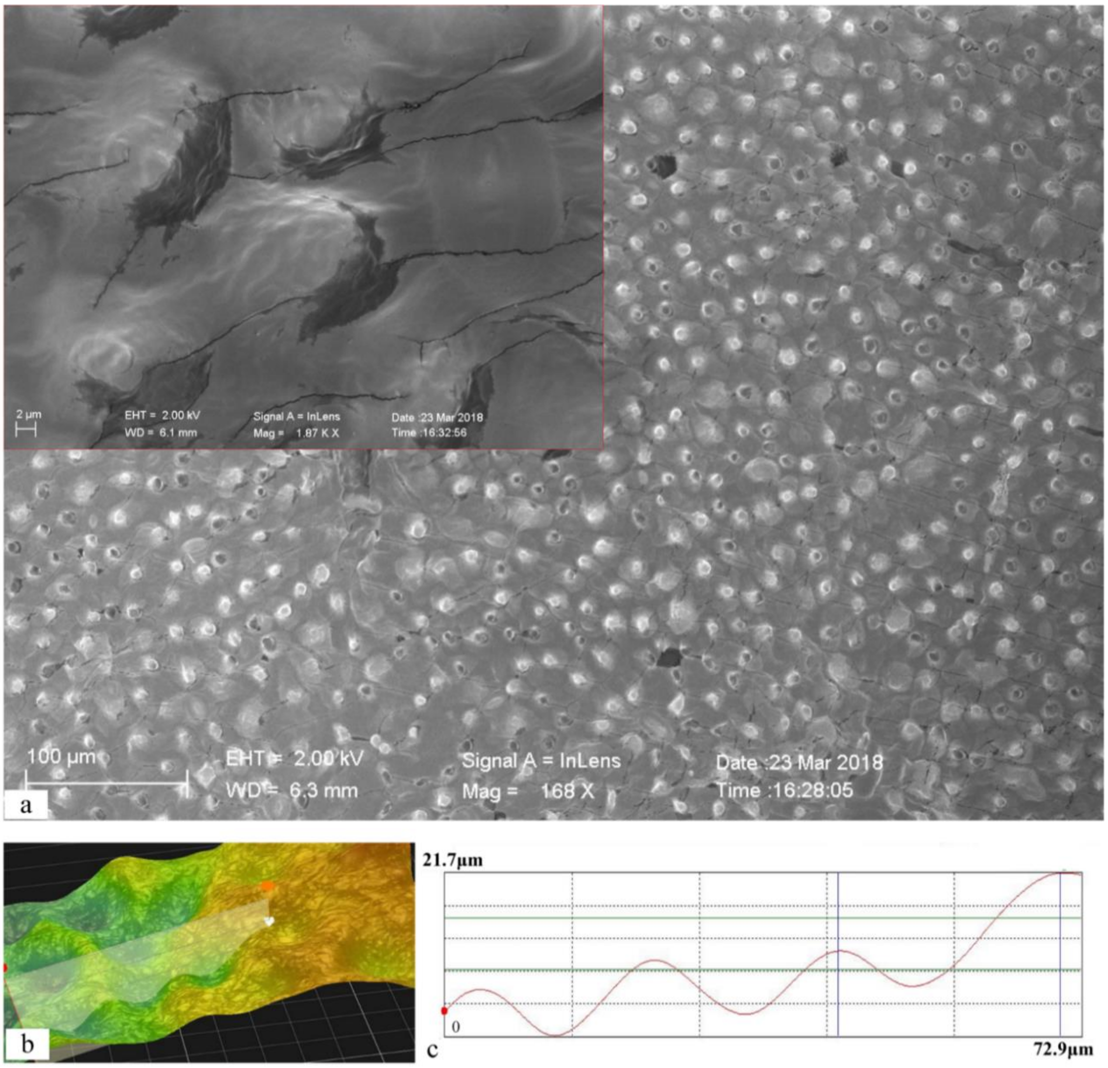

Figure S1 *Detailed observation of the lotus chip. (a) SEM image. (b) 3D microscopy reconstruction of the micro-pores. (c) Cross-sectional profile estimation of the lotus chip.*

2. Contact line on the sidewall of the bio-inspired micro-hole

According to the method of the chemically pre-patterned surfaces chip, the changing contact angle on the surface leads to pin the droplets to the three-phase contact line. We found that the splayed sidewall has a similar effect to the chemically pre-patterned surface chip, as shown in Figure S2a-b. When the liquid receding from the sidewall, the fluid shear stress occurs producing the droplets, as shown in Figure 2c-e.

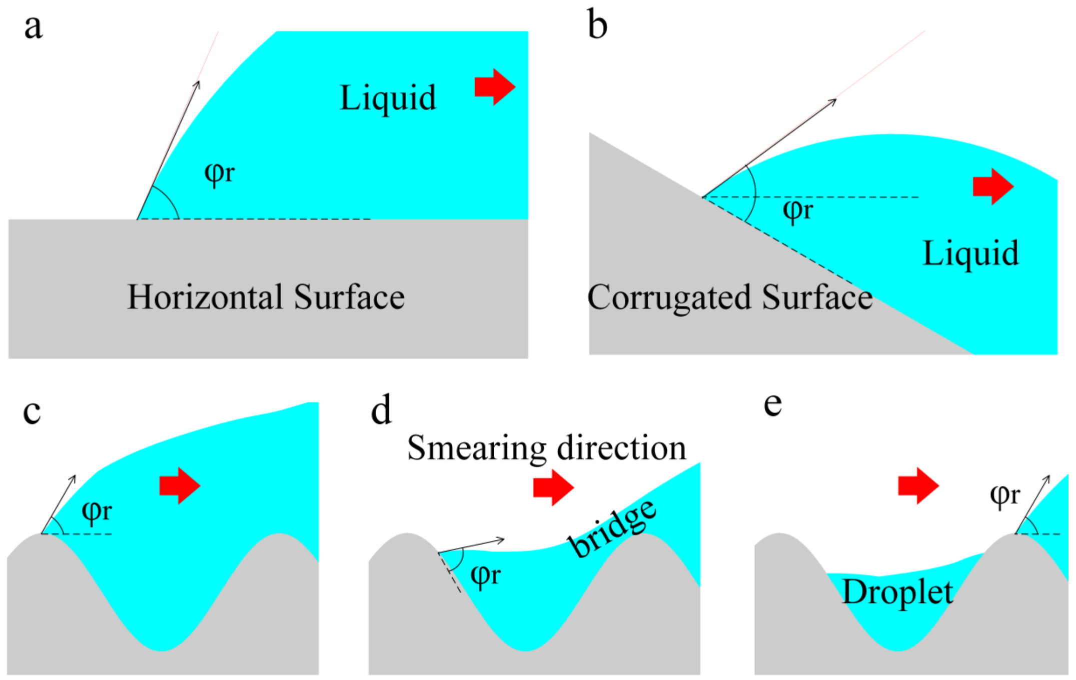

Figure S2 *Contact line on the splayed sidewall and generating droplet. (a) Contact line on the horizontal surface. (b) Contact line on the corrugated surface. (c-e) The process of generating droplets under the effect of fluid shear stress.*

3. Estimated the volume of the droplet

As shown in Figure S3, the one-quarter curve of the splayed sidewall is showing in a coordinate system. According to the experimental test, we measured the receding angles on the flat PDMS surface at 55°.

We estimate the volume of the droplet with and without considering the effect of the receding angle on the splayed sidewall. As shown in Figure S3a, we consider the effect of the receding angle φ_r to estimate the volume of the droplet. The volume of the red dotted line section is represented as the droplet volume. According to the geometric analysis, the approximate volume of the droplet in the different diameter of micro-holes are 1.0, 3.2, 14.08, 42.3 pL, respectively.

The estimated volume here is close to the experimental volume, but there is still a certain gap. This is because the liquid has a surface curvature resulting in the slight shift of the contact line position. The influence of the receding angle on the droplet volume becomes small, and we ignore the receding angle to estimate the droplet volume in the larger micro-hole. As shown in Figure S3b, the volume of the red dotted line section is represented as the droplet volume. According to the geometric analysis, the approximate volume of the droplet in the different diameter of micro-holes are 0.6, 1.9, 8.7, 26.6 pL, respectively.

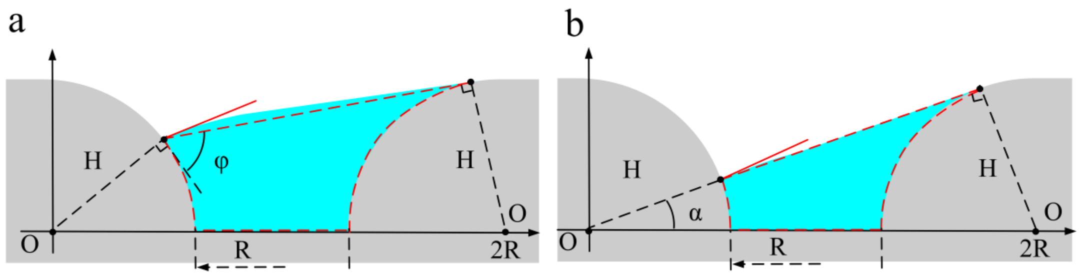

Figure S3 *The droplet volume estimation. (a) Schematic illustration of droplet volume estimation with considering the receding angle. (b) Schematic illustration of droplet volume estimation without considering the receding angle.*

4. The satellite droplets in one micro-hole

When the radius (R) is too large to the depth (H), it is easy to cause the appearance of satellite droplets in one micro-hole (Figure S4). The stability of the forming micro-droplet cannot be guaranteed, resulting in some satellite.

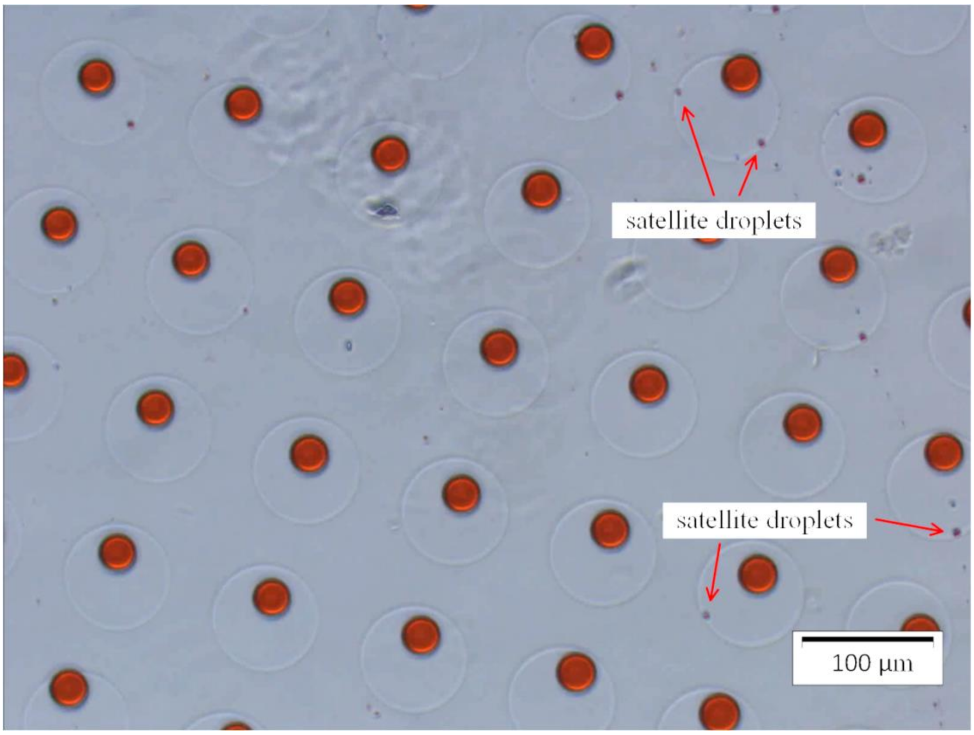

Figure S4 *The defects in forming micro-droplets (the depth is 10um, the bottom diameter is 80um).*

5. The droplet array formation on the chip with cylindrical microwells

To directly quantify the uniformity of droplets formed and ease of use with the gradual sidewall well design compared to the straight wall design, we used standard soft lithography to prepare a regular array of cylindrical micro-wells in PDMS. Briefly, we designed a mask to imitate the spatial period L = 22 μm). The mask sets the diameter of the wells to 20 μm. This mask pattern was used to prepare a 10 μm thick SU-8 2010 mold according to the manufacturer recommendations. The smearing process is similar to the above experiment sets. In Figure S5, the liquid “film” is actually cracked above the cylindrical micro-wells (similar behavior was also observed by other researches [36]).

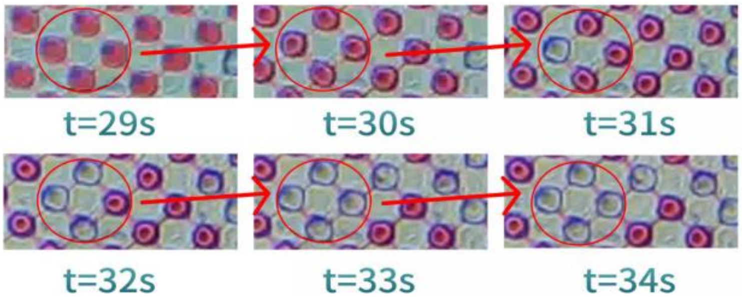

Figure S5 *Continuous dewetting of cylindrical micro-wells. The films start with a homogeneous red color indicating a constant thickness. After a few seconds, the center of the films becomes dark, indicating destructive optical interference due to film thinning.*

The experiments showed that only liquid films were forming on the surface of cylindrical holes and more than 40% of them broke within 8 seconds, which means at least 40% of such cylindrical holes cannot be filled with liquid by smearing.

6. The single-cell separation

The number distribution of bacteria in more micro-pores is showing in Figure S5.

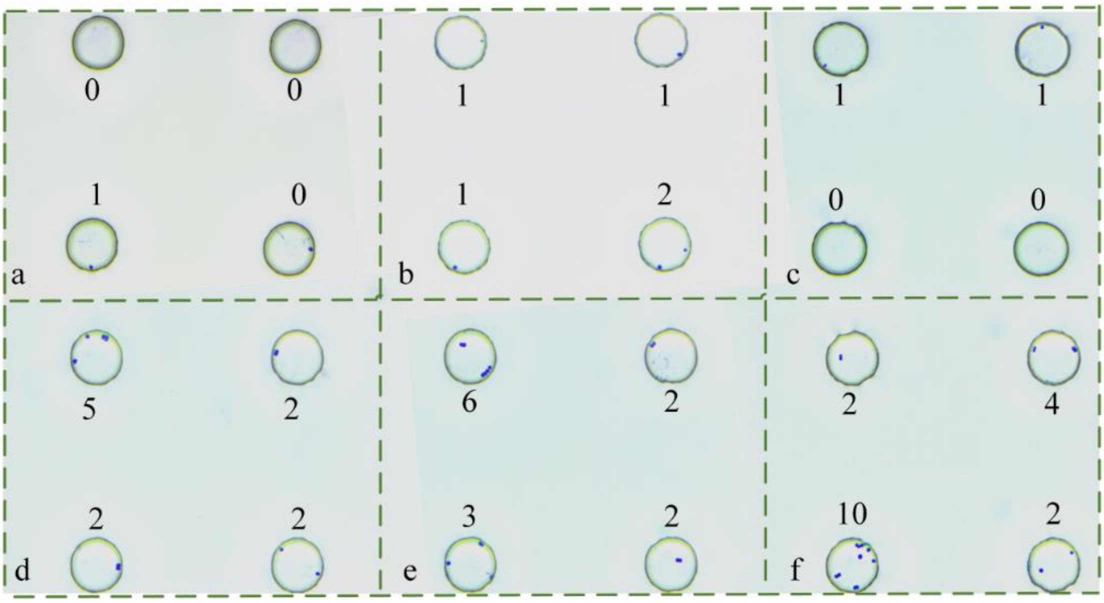

Figure S6 *The number distribution of bacteria in partial micro-holes (the depth is 10um, the bottom diameter is 10um). The numbers indicate the number of bacteria in each micro-hole, and the diameter of the hole is 30* μ*m.*

7. Videos about the process of micro-droplet array formation:

Video: In the video (captured by IX71, Olympus, Japan), the glass chip with bio-inspired micro-holes (the depth is 10um, the bottom diameter is 10um) is used to form the micro-droplet array by smearing. A micro-droplet array is easy to be got, as soon as the surface was gently smearing. After adding the oil, each droplet is wrapped into an approximate sphere, reducing evaporation for storing for a long time.
